# Design, synthesis and biological activity of hydroxybenzoic acid ester conjugates of phenazine-1-carboxylic acid

**DOI:** 10.1186/s13065-018-0478-2

**Published:** 2018-11-01

**Authors:** Xiang Zhu, Linhua Yu, Min Zhang, Zhihong Xu, Zongli Yao, Qinglai Wu, Xiaoying Du, Junkai Li

**Affiliations:** 1 Hubei Collaborative Innovation Centre for Grain Industry, Yangtze University, Jingmi Road 88, Jingzhou, 434025 China; 2 School of Agriculture, Yangtze University, Jingmi Road 88, Jingzhou, 434025 China; 3 Engineering Research Center of Ecology and Agricultural Use of Wetland, Ministry of Education, Yangtze University, Jingmi Road 88, Jingzhou, 434025 China

**Keywords:** Phenazine-1-carboxylic acid, Synthesis, Biological activity, Salicylic acid

## Abstract

We prepared 16 novel hydroxybenzoic acid ester conjugates of phenazine-1-carboxylic acid (PCA) and investigated their biological activity. Most of the synthesized conjugates displayed some level of fungicidal activities in vitro against five phytopathogenic fungi. Nine conjugates **5b**, **5c**, **5d**, **5e**, **5h**, **5i**, **5m**, **5n** and **5o** (EC_50_ between 3.2 μg/mL and 14.1 μg/mL) were more active than PCA (EC_50_ 18.6 μg/mL) against *Rhizoctonia solani*. Especially conjugate **5c** showed the higher fungicidal activity against *Rhizoctonia solani* which is 6.5-fold than PCA. And the results of the bioassay indicated that the fungicidal activity of conjugates was associated with their LogP, and the optimal LogP values of the more potent fungicidal activities within these conjugates ranged from 4.42 to 5.08. The systemic acquired resistance induced by PCA–SA ester conjugate **5c** against rice sheath blight disease in rice seedlings was evaluated. The results revealed that PCA–SA ester conjugate **5c** retained the resistance induction activity of SA against rice sheath blight.

**Electronic supplementary material:**

The online version of this article (10.1186/s13065-018-0478-2) contains supplementary material, which is available to authorized users.

## Background

**Fig. 1:**
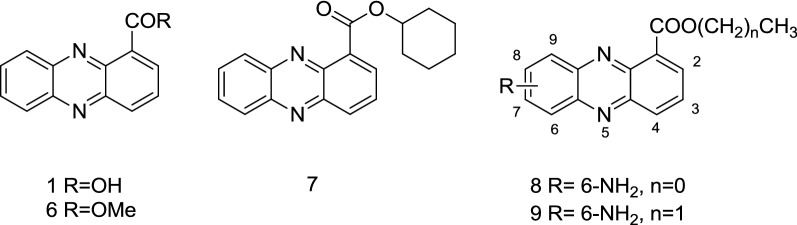
The structures of PCA and its derivatives

Phenazine-1-carboxylic acid (PCA) (**1**, Fig. 1) is a secondary metabolite isolated from *Pseudomonas*, *Streptomycetes*, and a few other bacterial genera from soil or marine habitats [1–5]. The biological properties of PCA includes antimicrobial [6–9] antiviral [7], antitumorigenic [8–12] antitubercular and antileukemic activities [13, 14]. In China, PCA has been registered as a biofungicide against rice sheath blight caused by *Rhizoctonia solani*, and it is noted for its high efficacy, low toxicity, environmental friendliness and enhancement of crop production [15–18]. PCA is also an important precursor for the biosynthesis of ester derivatives [1, 19], some of which show higher fungicidal activity against several phytopathogenic fungi. For instance, compound **6** (Fig. 1) isolated from *Pseudomonas*, was a more effective derivative against *Alternaria alternata* and *R. solani* than PCA [5]. As reported, some synthetic phenazine-1-carboxylate derivatives prepared by chemical modification of the carboxyl group with various alkyl alcohols exhibit strong fungicidal activity against *Pyricularia oryzae*, and in particular the inhibition of derivative **7** was 100% complete at 8.3 μg/mL [20]. Recently, a series of novel aminophenazine-1-carboxylate derivatives were synthesized and evaluated against five fungi [21], and the results of bioassay showed that compounds **8** and **9** (Fig. 1) could exhibited strong activity against *P. piricola* with EC_50_ values of 3.00 μg/mL and 4.44 μg/mL respectively, which were both lower than that of PCA.

**Fig. 2:**
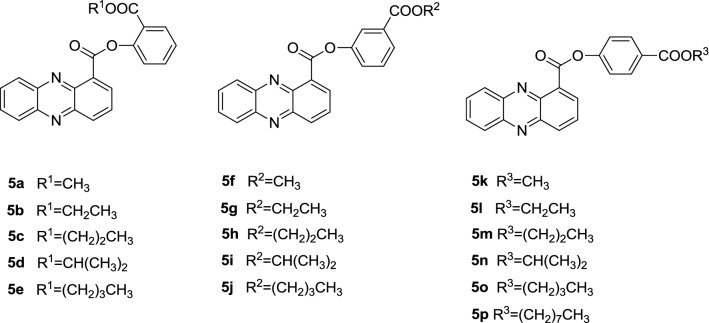
The structures of PCA–salicylic acid ester conjugates (**5a**–**5e**), PCA-3-hydroxybenzoic acid ester conjugates (**5f**–**5j**) and PCA-*p*-hydroxybenzoic acid ester conjugates (**5k**–**5p**)

Salicylic acid (SA) (Fig. 2), also known as *o*-hydroxybenzoic acid which is one of the three isomers of hydroxybenzoic acid, is an important plant growth regulator playing a role in the hypersensitive reaction (HR) and acts as an endogenous signal responsible for inducing systemic acquired resistance in plants [22, 23]. The plants treated with salicylic acid or its derivatives may be able to resist infection by various plant pathogens [24–26]. Hydroxybenzoate esters, which are widely used in medicine, foods and cosmetics, have been reported to have various biological activities, such as antimicrobial [27–29] antiviral [30, 31], anti-inflammatory and nematicidal activities [32], among others. Accordingly, hydroxybenzoate esters with multiple bioactive chemical structures, have drawn wide attention in the biological and pharmacological fields.

**Fig. 3:**
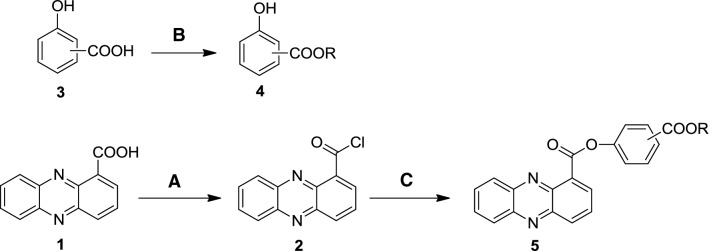
Synthetic route of target compounds. Reagents and conditions: **a** oxalyl chloride, CH_2_Cl_2_, DMF, reflux, 8 h; **b** alcohol, reflux, overnight; **c** hydroxybenzoic acid ester, CH_2_Cl_2_, room temperature to reflux, 2 h

In this research, considering the potential biological activity of phenazine-1-carboxylic derivatives and that there have been few published studies on the biological activity of phenazine-1-carboxylic phenolic esters, we designed and synthesized 16 novel phenolic ester derivatives of phenazine-1-carboxylic acid (Fig. 2) by a simple esterification reaction of PCA and three types of hydroxybenzoic acids. To enhance the lipophilic properties of the these conjugates, hydroxybenzoic acids were derivatized to its ester with the corresponding CH_3_(CH_2_)nOH. The synthetic route of conjugates **5a**–**5p** is described in Fig. 3. All these conjugates were evaluated for their fungicidal activity against five phytopathogenic fungi in vitro. Furthermore, the systemic acquired resistance of the most active PCA–SA ester conjugate **5c** against rice sheath blight disease was also investigated in rice plants.

## Results and discussion

### Chemistry

As shown in Fig. 3, three types of hydroxybenzoate esters (**4**) were first synthesized by a simple esterification reaction with 2-hydroxybenzoic acid, 3-hydroxybenzoic acid or 4-hydroxybenzoic acid as the starting materials. Then treatment of PCA with oxalyl chloride at the reflux temperature in CH_2_Cl_2_ solution afforded intermediate **2** after the evaporation of CH_2_Cl_2_. The target compound **5a** was synthesized by adding intermediate **2** to compound **4a** in CH_2_Cl_2_ solution, stirred at room temperature for 2 h. PCA–salicylic acid ester conjugates (**5a**–**5e**), PCA-3-hydroxybenzoic acid ester conjugates (**5f**–**5j**) and PCA-*p*-hydroxybenzoic acid ester conjugates (**5k**–**5p**) were synthesized by this method.

**Fig. 4:**
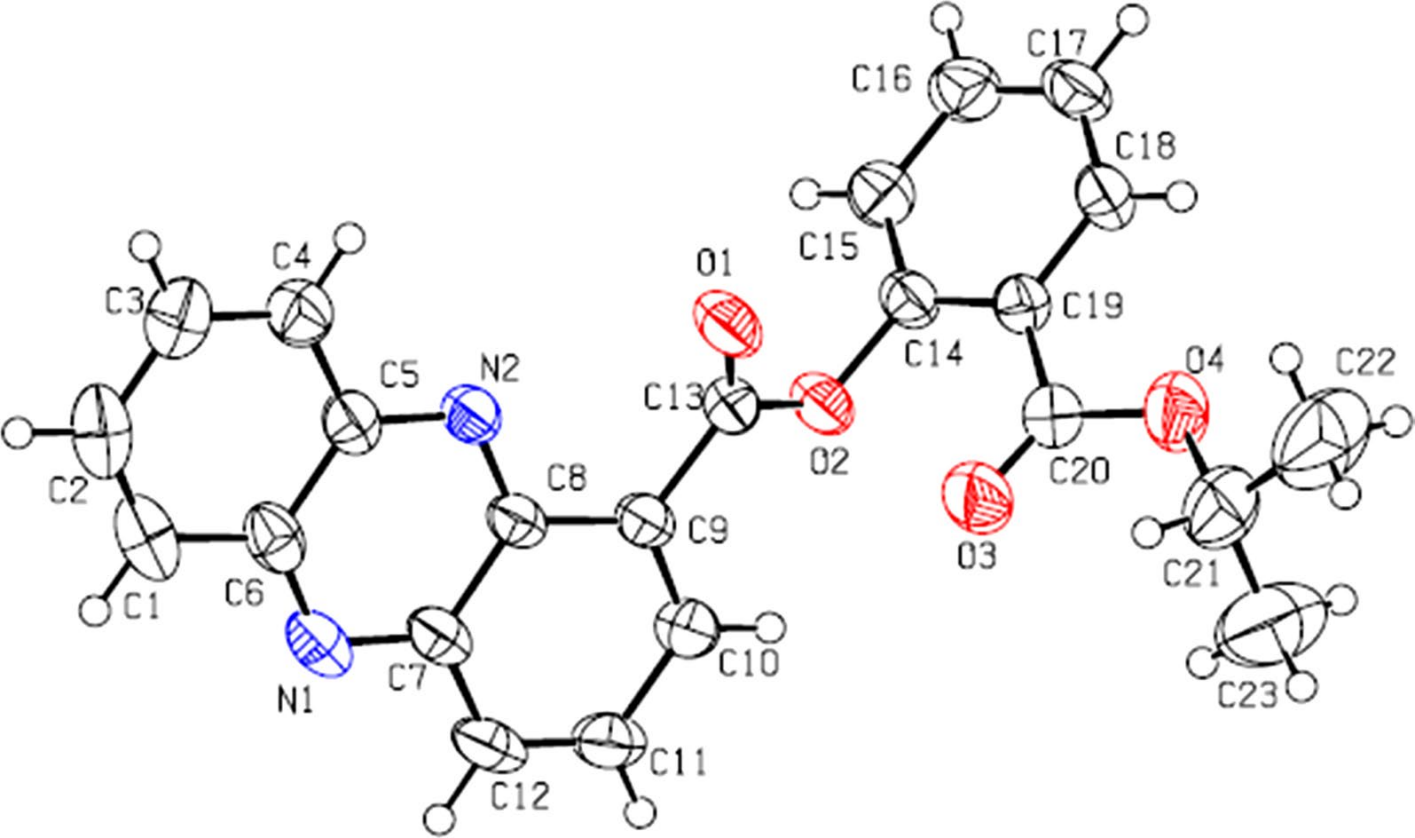
The crystal structure of conjugate **5d**

The structures of all conjugates were characterized by ^1^H NMR and high resolution mass spectroscopy (HRMS) analyses, and the representative conjugate **5d** was confirmed by the X-ray crystallographic analysis. The molecular structure of **5d** is shown in Fig. 4. The crystal data for **5d**: triclinic, space group P2_1_/c, a = 18.130 (3) Å, b = 12.258 (2) Å, c = 8.6490 (14) Å, a = 90°, b = 96.224 (3)°, g = 90°, V = 1910.7 (6) Å3, Z = 4, T = 297 (2) K, μ (Mο) = 0.093 mm^−1^, D_calcd._ = 1.343 Mg/m^3^, 14,129 reflections measured (1.130 ≤ 2Ɵ ≤ 26.000°), 3755 unique (R (int) = 0.0316) which were used in all calculations. The final *R*_1_ was 0.0408 (I > 2 sigma (I)) and *wR*_2_ was 0.1162. Crystallographic data have been deposited with the Cambridge Crystallographic Data Centre, and the deposition number was CCDC 1563918 (Additional file 1).

### Fungicidal activities

All novel conjugates (**5a**–**5p**) were primarily screened in vitro against five phytopathogenic fungi, *R. solani*, *A. solani*, *Fusarium oxysporum*, *Fusaium graminearum* and *P. oryzae*, with PCA as a control. The results of the preliminary bioassay are shown in Table 1. We found that most of conjugates (**5a**–**5p**) showed low activities against *A*. *solani*, *F*. *oxysporum*, *F*. *graminearum* and *P*. *oryzae* Cavara at a concentration of 50 μg/mL, while most conjugates (**5a**–**5p**) exhibited high activity against *R. solani* at that rate. The inhibitory activity of **5c**, **5e**, **5i** and **5m** was 100%, higher than PCA at 86.2%. To more closely examine preliminary structure–activity relationships (SARs), the conjugates (**5a**–**5p**) were selected for assessment of EC_50_ values against *Rhizoctonia solani*.

**Table 1:** Fungicidal activity of compounds **5a**–**5p** against five plant fungi in vitro at 50 μg/mL (inhibition rate/%)

Compd.	*R. solani*	*A. solani*	*F. oxysporum*	*F. graminearum*	*P. oryzae*
**5a**	66.2 ± 1.5	11.7 ± 0.5	13.1 ± 0.6	7.3 ± 0.9	34.5 ± 0.9
**5b**	91.6 ± 0.8	30.3 ± 1.6	15.7 ± 1.3	15.9 ± 2.6	32.8 ± 0.0
**5c**	100.0 ± 0.0	13.0 ± 2.3	12.4 ± 0.8	6.5 ± 0.2	37.0 ± 2.7
**5d**	93.5 ± 0.6	12.4 ± 0.9	31.4 ± 2.9	12.3 ± 1.3	27.0 ± 1.2
**5e**	93.1 ± 0.9	15.1 ± 0.6	9.8 ± 0.3	10.9 ± 0.6	27.0 ± 3.9
**5f**	37.3 ± 1.2	45.5 ± 0.3	35.3 ± 3.4	13.0 ± 0.9	72.3 ± 0.0
**5** **g**	41.2 ± 0.8	21.3 ± 1.3	16.3 ± 0.9	11.6 ± 2.7	49.6 ± 2.6
**5** **h**	93.2 ± 0.3	15.1 ± 0.5	9.8 ± 0.5	10.9 ± 3.4	27.0 ± 0.9
**5i**	100.0 ± 0.0	28.9 ± 1.8	18.3 ± 2.7	10.1 ± 0.5	45.4 ± 1.2
**5j**	45.1 ± 1.0	17.2 ± 2.5	13.1 ± 0.5	11.6 ± 1.9	39.6 ± 1.5
**5k**	33.9 ± 0.9	18.6 ± 0.3	11.1 ± 0.6	8.7 ± 3.5	39.6 ± 3.7
**5l**	42.4 ± 1.2	19.3 ± 0.9	13.7 ± 1.1	13.0 ± 4.4	45.4 ± 0.9
**5m**	100.0 ± 0.0	24.1 ± 1.5	15.7 ± 1.6	10.9 ± 0.8	39.6 ± 0.2
**5n**	98.3 ± 0.2	26.2 ± 0.9	13.7 ± 1.5	8.7 ± 4.6	41.2 ± 0.9
**5o**	93.0 ± 0.2	15.1 ± 0.5	9.8 ± 2.9	10.9 ± 3.3	27.0 ± 0.8
**5p**	44.5 ± 1.2	18.6 ± 0.9	13.7 ± 0.9	0 ± 0.0	34.5 ± 4.9
**PCA**	86.2 ± 0.9	85.2 ± 1.2	83.5 ± 1.9	86.1 ± 1.9	92.0 ± 2.7

Each treatment had three replicates (Mean ± SD). The phenazine-1carboxylic acid (PCA) was used as the positive control

The EC_50_ values against *Rhizoctonia solani* for all conjugates are presented in Table 2. The results showed that nine conjugates (**5b**, **5c**, **5d**, **5e**, **5h**, **5i**, **5m**, **5n** and **5o**) with EC_50_ values between 3.2 and 14.1 μg/mL exhibited more potent fungicidal activity against *Rhizoctonia solani* than PCA (EC_50_ = 18.6 μg/mL). In particular, conjugate **5c** with highest fungicidal activity was 6.5-fold more active than PCA.

**Table 2:** EC_50_ values against *Rhizoctonia solani* and octanol–water partition coefficient of conjugates **5a**–**5p**

Compd.	EC_50_ (μg/mL)	Toxicity index	LogP^1^
**5a**	48.3	0.43	3.84
**5b**	14.1	1.48	4.42
**5c**	3.2	6.50	4.72
**5d**	9.5	2.19	4.75
**5e**	12.8	1.63	5.08
**5f**	96.3	0.22	3.91
**5g**	68.6	0.30	4.34
**5h**	9.5	2.19	4.81
**5i**	4.9	4.24	4.77
**5j**	56.9	0.37	5.02
**5k**	138.4	0.15	3.92
**5l**	70.5	0.30	4.37
**5** **m**	4.5	4.62	4.86
**5n**	5.6	3.71	4.75
**5o**	11.8	1.76	5.05
**5p**	70	0.30	6.46
**PCA**	18.6	1.00	1.59

^1^Partition coefficient ‘‘LogP’’ values were calculated using the ALOGPS 2.1 program

**Fig. 5:**
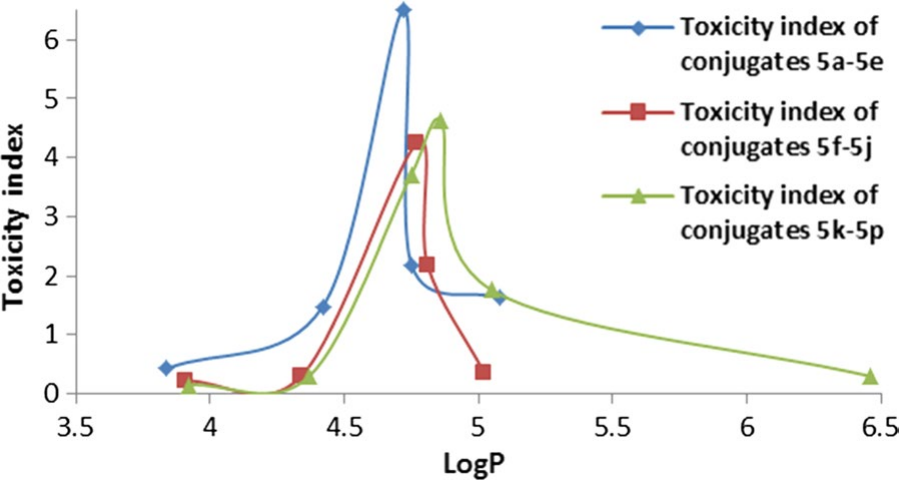
The toxicity index of conjugates **5a**–**5p**

The recent study on fungicidal mechanism of PCA indicate that, PCA will promote cell produces poisonous hydroxyl radical and disrupt the normal homeostasis of redox in cells after entering cells through cell walls and cell membranes [19, 33]. It means that a PCA analog with suitable polarity and hydrophobicity can pass through the cell membranes of pathogenic bacteria and fungi more easily and exhibit higher biological activity. As can be seen from Table 2, the fungicidal activities of conjugates were associated with their LogP values. Accordingly, we constructed a mathematical model that described the LogP of conjugates that might be expected to produce high or low levels of fungicidal activity. From Fig. 5, with increasing LogP values, the fungicidal activities of conjugates were also observed to increase. For instance, the LogP values of PCA–salicylic acid ester conjugates were ranked as follows: **5a **< **5b **< **5c**, and the fungicidal activity of conjugates also showed the same ranking. However, the conjugates that exceeded a certain level of LogP values (> 4.72) had decreased fungicidal activity. For instance, the LogP values of PCA–salicylic acid ester conjugates were ranked **5c **<** 5d **<** 5e**, but the fungicidal activity of conjugates were ranked **5c **>** 5d **> **5e**. The same trends also applied to the PCA-3-hydroxybenzoic acid ester conjugates (**5f**–**5j**) and the PCA-*p*-hydroxybenzoic acid ester conjugates (**5k**–**5p**). Through the above analysis, we found that the LogP values of the more potent fungicidal activity within these three types of conjugates ranged from 4.42 to 5.08. Furthermore, conjugates where phenolic ester groups were substituted at different positions did not greatly affect their fungicidal activity.

### Systemic acquired resistance

**Fig. 6:**
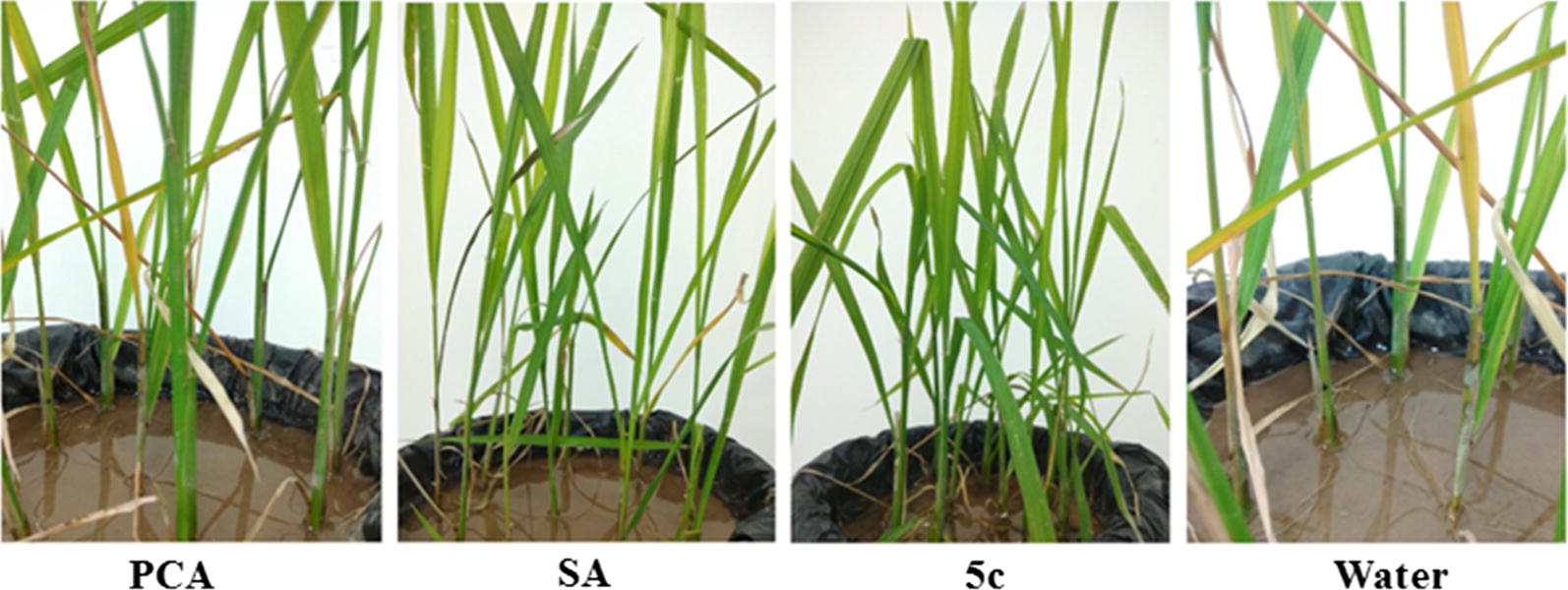
Protection of rice plants against *Rhizoctonia solani* by a foliar spray with 200 μmol/L PCA (**PCA**), 200 μmol/L SA (**SA**), 200 μmol/L of conjugate **5c** (**5c**) and distilled water (water) 14 days after inoculation with *Rhizoctonia solani* conidial suspension (10^5^ spore/mL)

To evaluate the level of systemic acquired resistance induced by PCA–SA ester conjugates, the disease reduction of the most active PCA–SA ester conjugate **5c** was investigated against rice sheath blight disease on rice seedlings following Makandar and others [34, 35]. The results of the study indicated that inoculation with conidia of *Rhizoctonia solani* onto rice plants treated with SA and conjugate **5c** resulted in fewer lesions per leaf sheath as well as reduced blighted leaf area as compared to control plants only receiving distilled water treatment (Fig. 6). Spray treatment with SA and PCA–SA ester conjugate **5c** induced resistance to sheath blight disease in rice plants, significantly reducing rice sheath blight disease in rice plants. Compared with the treatments of PCA and water control, combined SA and conjugate **5c** treatments had higher induction effects, at 31.0% and 57.0% respectively (Table 3).

**Table 3:** Induced resistance of rice to rice sheath blight by different inducers treatment

Inducers treatment	Disease (%)	Induced effect (%)
A (PCA)	47.9	12.59
B (SA)	37.3	31.97
C (conjugate **5c**)	23.6	56.98
D (water)	54.8	–

At present, there is extensive research on possible structure–activity relationship of SA and its derivatives for induction of systemic acquired resistance. Safari assessed the potential of some chemical inducers of systemic acquired resistance (SAR) to reduce *Alternaria* leaf spot disease on tomato in glasshouse trials [26]. The results indicated that, among the salicylate derivatives, the biochemical activators containing electron donating groups are more suitable for inducing disease resistance in tomato crop. Also the structure relationship of 47 mono-substituted and multi-substituted salicylate derivatives with respect to their effects on disease resistance to tobacco mosaic virus and pathogenesis-related protein (PR1) accumulation were evaluated [25]. In this study, using this characteristic of SA, we demonstrated that PCA–SA ester conjugate **5c** retained the resistance induction activity of SA against rice sheath blight and had higher induced resistance than SA. However, the relationship between the structures of PCA–SA ester conjugates described here and their induced activities needs further investigation, as well as the mode of action.

## Experimental

### Chemicals and instruments

All chemicals and solvents were obtained from commercial suppliers and were used without further purification. The melting points were determined on a WRR melting point apparatus (Shanghai Jingke Industrial Co. Ltd., PR China) and were uncorrected. Thin-layer chromatography (TLC) was performed on silica gel 60 F254 (Qingdao Marine Chemical Ltd., P. R. China). Column chromatography (CC) purification was performed over silica gel (200–300 mesh, Qingdao Marine Chemical Ltd.). ^1^H NMR spectrum were recorded in CDCl_3_ solution on a Bruker 600 MHz spectrometer (Bruker Co., Switzerland), using tetramethylsilane (TMS) as an internal standard, and chemical shift values (δ) were given in parts per million (ppm). The following abbreviations were used to designate chemical shift multiplicities: s = singlet, d = doublet, t = triplet, q = quartet, m = multiple. MS data were obtained using a APEX IV Fourier-transform mass spectrometry (Bruker).

### Synthesis of hydroxybenzoic acid esters

The compound 2-hydroxybenzoic acid (15 mmol) and its corresponding alcohol (30 mL) were added into a 50 mL round-bottom flask, and cooled at 0 °C. An aliquot of 2 mL of 98% H_2_SO_4_ was slowly added. The reaction was stirred at reflux temperature for 12 h and monitored by thin-layer chromatography (TLC) until the 2-hydroxybenzoic acid was completely consumed. The mixture was evaporated under vacuum, neutralized with water and 5% NaHCO_3_ aqueous solution, extracted by ether 3 times, dried over Na_2_SO_4_, concentrated in vacuum, and used in next step without purification. The compounds 3-hydroxybenzoic acid esters and *p*-hydroxybenzoic acid esters were also synthesized by this method.

### Synthesis of phenazine-1-carbonyl chloride

Phenazine-1-carboxylic acid (10 mmol) and *N*,*N*-dimethylformamide (0.1 mmol) were added in 30 mL of dry CH_2_Cl_2_, and cooled at 0 °C. A solution of 15 mmol of oxalyl chloride in 20 mL of dry CH_2_Cl_2_ was then slowly added. The reaction was stirred at reflux temperature for 12 h, then cooled to room temperature and evaporated under vacuum. The residue was dissolved in 10 mL of dry CH_2_Cl_2_ and used in next step without purification.

### General procedure for hydroxybenzoic acid ester conjugates of phenazine-1-carboxylic acid **5a**–**5p**

Phenazine-1-carbonyl chloride (10 mmol) dissolved in 10 mL of dry CH_2_Cl_2_ was added dropwise to a solution of compound 2-hydroxybenzoic acid methyl ester (10 mmol), and triethylamine (12 mmol) as the attaching acid agent in CH_2_Cl_2_, The mixture was stirred at room temperature for 4 h until the reaction was complete (indicated by TLC), then quenched with water and 5% Na_2_CO_3_ aqueous solution, dried over Na_2_SO_4_, filtered and concentrated in vacuum. The obtained crude extract was purified by recrystallizing from the solution of EtOAc-DCM (1:1) to give pure conjugate **5a**. Conjugates **5b**–**5p** were also synthesized by this method.

### 2-(Methoxycarbonyl)phenyl phenazine-1-carboxylate (**5a**)

Yellow solid; yield: 89.5%; m.p. 141–142 °C; ^1^H-NMR (600 MHz, CDCl_3_) δ: 8.69 (d, J = 7.2 Hz, 1H), 8.49 (d, J = 8.8 Hz, 1H), 8.36 (dd, J = 6.0, 3.6 Hz, 1H), 8.28 (dd, J = 6.6, 3.6 Hz, 1H), 8.14 (dd, J = 7.8, 1.2 Hz, 1H), 7.98 (dd, J = 8.4, 7.2 Hz, 1H), 7.94–7.87 (m, 2H), 7.74–7.68 (m, 1H), 7.51 (d, J = 7.8 Hz, 1H), 7.43 (t, J = 7.8 Hz, 1H), 3.86 (s, 3H). HRMS calcd for C_21_H_14_N_2_O_4_ [M+H]^+^: 359.1026, found 359.1027.

### 2-(Ethoxycarbonyl)phenyl phenazine-1-carboxylate (**5b**)

Yellow solid; yield: 92.3%; m.p. 143–144 °C; ^1^H-NMR (600 MHz, CDCl_3_) δ: 8.74–8.69 (m, 1H), 8.48 (dd, J = 8.4, 1.2 Hz, 1H), 8.36 (dd, J = 6.6, 3.6 Hz, 1H), 8.28 (dd, J = 6.6, 3.6 Hz, 1H), 8.14 (dd, J = 7.8, 1.2 Hz, 1H), 7.98 (dd, J = 8.4, 7.2 Hz, 1H), 7.93–7.86 (m, 2H), 7.74–7.66 (m, 1H), 7.50 (d, J = 7.8 Hz, 1H), 7.42 (t, J = 7.8 Hz, 1H), 4.33 (q, J = 7.2 Hz, 2H), 1.27 (t, J = 7.2 Hz, 3H). HRMS calcd for C_22_H_16_N_2_O_4_ [M+H]^+^: 373.1183, found 373.1182.

### 2-(Propoxycarbonyl)phenyl phenazine-1-carboxylate (**5c**)

Yellow solid; yield: 97.5%; m.p. 102–103 °C; ^1^H-NMR (600 MHz, CDCl_3_) δ 8.72 (dd, *J* = 6.6, 1.2 Hz, 1H), 8.48 (dd, *J* = 8.4, 1.2 Hz, 1H), 8.40–8.31 (m, 1H), 8.32–8.21 (m, 1H), 8.14 (dd, *J* = 7.8, 1.8 Hz, 1H), 7.98 (dd, *J* = 8.4, 7.2 Hz, 1H), 7.94–7.79 (m, 2H), 7.73–7.59 (m, 1H), 7.51 (d, *J* = 7.2 Hz, 1H), 7.43 (dd, *J* = 11.4, 4.2 Hz, 1H), 4.23 (t, *J* = 6.6 Hz, 2H), 1.74–1.41 (m, 2H), 0.92 (t, *J* = 7.2 Hz, 3H). HRMS calcd for C_23_H_18_N_2_O_4_ [M+H]^+^: 387.1339, found 387.1338.

### 2-(Isopropoxycarbonyl)phenyl phenazine-1-carboxylate (**5d**)

Yellow solid; yield: 90.5%; m.p. 125–126 °C; ^1^H-NMR (600 MHz, CDCl_3_) δ 8.73 (d, *J* = 6.6 Hz, 1H), 8.48 (d, *J* = 8.4 Hz, 1H), 8.36 (dd, *J* = 6.6, 3.6 Hz, 1H), 8.27 (dd, *J* = 6.6, 3.6 Hz, 1H), 8.12 (dd, *J* = 7.8, 1.2 Hz, 1H), 7.98 (dd, *J* = 8.4, 7.2 Hz, 1H), 7.93–7.86 (m, 2H), 7.71–7.66 (m, 1H), 7.50 (d, *J* = 7.8 Hz, 1H), 7.41 (t, *J* = 7.8 Hz, 1H), 5.30–5.30 (m, 1H), 1.27 (d, *J* = 6.6 Hz, 6H). HRMS calcd for C_23_H_18_N_2_O_4_ [M+H]^+^: 387.1339, found 387.1340.

### 2-(Butoxycarbonyl)phenyl phenazine-1-carboxylate (**5e**)

Yellow solid; yield: 94.1%; m.p. 89–90 °C; ^1^H-NMR (600 MHz, CDCl_3_) δ 8.72 (dd, *J* = 6.9, 1.4 Hz, 1H), 8.48 (dd, *J* = 8.7, 1.4 Hz, 1H), 8.39–8.34 (m, 1H), 8.30–8.25 (m, 1H), 8.13 (dd, *J* = 7.9, 1.7 Hz, 1H), 7.98 (dd, *J* = 8.4, 6.6 Hz, 1H), 7.93–7.86 (m, 2H), 7.72–7.67 (m, 1H), 7.51 (dd, *J* = 7.8, 1.2 Hz, 1H), 7.47–7.37 (m, 1H), 4.27 (t, *J* = 6.7 Hz, 2H), 1.72–7.57 (m, 2H), 1.42–1.31 (m, 2H), 0.84 (t, *J* = 7.2 Hz, 3H). HRMS calcd for C_24_H_20_N_2_O_4_ [M+H]^+^: 401.1496, found 401.1497.

### 3-(Methoxycarbonyl)phenyl phenazine-1-carboxylate (**5f**)

Yellow solid; yield: 95.0%; m.p. 120–121 °C; ^1^H-NMR (600 MHz, CDCl_3_) δ 8.48 (t, *J* = 7.2 Hz, 2H), 8.39–8.34 (m, 1H), 8.30–8.25 (m, 1H), 8.14 (s, 1H), 8.03 (d, *J* = 7.8 Hz, 1H), 7.98–7.89 (m, 3H), 7.70–7.66 (m, 1H), 7.59 (t, *J* = 7.8 Hz, 1H), 3.98 (s, 3H). HRMS calcd for C_21_H_14_N_2_O_4_ [M+H]^+^: 359.1026, found 359.1027.

### 3-(Ethoxycarbonyl)phenyl phenazine-1-carboxylate (**5g**)

Yellow solid; yield: 96.5%; m.p. 109–110 °C; ^1^H-NMR (600 MHz, CDCl_3_) δ 8.55–8.41 (m, 2H), 8.39–8.30 (m, 1H), 8.31–8.24 (m, 1H), 8.14 (s, 1H), 8.04 (d, *J* = 7.8 Hz, 1H), 7.98–7.85 (m, 3H), 7.67 (d, *J* = 7.8 Hz, 1H), 7.59 (t, *J* = 7.8 Hz, 1H), 4.44 (q, *J* = 7.2 Hz, 2H), 1.44 (t, *J* = 7.2 Hz, 3H). HRMS calcd for C_22_H_16_N_2_O_4_ [M+H]^+^: 373.1183, found 373.1182.

### 3-(Propoxycarbonyl)phenyl phenazine-1-carboxylate (**5h**)

Yellow solid; yield: 95.2%; m.p. 87–88 °C; ^1^H-NMR (600 MHz, CDCl_3_) δ 8.51–8.43 (m, 2H), 8.38–8.32 (m, 1H), 8.27 (dd, *J* = 6.0, 4.2 Hz, 1H), 8.13 (s, 1H), 8.04 (d, *J* = 7.8 Hz, 1H), 7.97–7.86 (m, 3H), 7.67 (dd, *J* = 7.8, 1.2 Hz, 1H), 7.59 (t, *J* = 7.8 Hz, 1H), 4.34 (t, *J* = 6.6 Hz, 2H), 1.88–1.82 (m, 1H), 1.06 (t, *J* = 7.8 Hz, 3H). HRMS calcd for C_23_H_18_N_2_O_4_ [M+H]^+^: 387.1339, found 387.1340.

### 3-(Butoxycarbonyl)phenyl phenazine-1-carboxylate (**5i**)

Yellow solid; yield: 95.5%; m.p. 97–98 °C; ^1^H-NMR (600 MHz, CDCl_3_) δ 8.47 (d, *J* = 7.8 Hz, 2H), 8.39–8.31 (m, 1H), 8.29–8.23 (m, 1H), 8.15–8.09 (m, 1H), 8.03 (d, *J* = 7.8 Hz, 1H), 7.97–7.85 (m, 3H), 7.66 (dd, *J* = 7.8, 2.0 Hz, 1H), 7.58 (t, *J* = 7.8 Hz, 1H), 5.37–5.25 (m, 1H), 1.41 (d, *J* = 6.6 Hz, 6H). HRMS calcd for C_23_H_18_N_2_O_4_ [M+H]^+^: 387.1339, found 387.1340.

### 3-(Butoxycarbonyl)phenyl phenazine-1-carboxylate (**5j**)

Yellow solid; yield: 95.2%; m.p. 87–88 °C; ^1^H-NMR (600 MHz, CDCl_3_) δ 8.48 (dd, *J* = 7.8, 3.6 Hz, 2H), 8.39–8.33 (m, 1H), 8.31–8.25 (m, 1H), 8.12 (s, 1H), 8.04 (d, *J* = 7.8 Hz, 1H), 7.98–7.90 (m, 3H), 7.67 (dd, *J* = 7.8, 2.4 Hz, 1H), 7.59 (t, *J* = 7.8 Hz, 1H), 4.39 (t, *J* = 6.6 Hz, 2H), 1.83–1.77 (m, 2H), 1.56–1.48 (m, 2H), 1.01 (t, *J* = 7.2 Hz, 3H). HRMS calcd for C_24_H_20_N_2_O_4_ [M+H]^+^: 401.1496, found 401.1495.

### 4-(Methoxycarbonyl)phenyl phenazine-1-carboxylate (**5k**)

Yellow solid; yield: 95.0%; m.p. 164–165 °C; ^1^H-NMR (600 MHz, CDCl_3_) δ 8.54–8.43 (m, 2H), 8.37–8.33 (m, 1H), 8.31–8.26 (m, 1H), 8.24–8.18 (m, 2H), 7.97–7.90 (m, 3H), 7.57–7.51 (m, 2H), 3.97 (s, 3H). HRMS calcd for C_21_H_14_N_2_O_4_ [M+H]^+^: 359.1026, found 359.1025.

### 4-(Ethoxycarbonyl)phenyl phenazine-1-carboxylate (**5l**)

Yellow solid; yield: 98.1%; m.p. 123–125 °C; ^1^H-NMR (600 MHz, CDCl_3_) δ 8.51–8.46 (m, 2H), 8.38–8.32 (m, 1H), 8.31–8.26 (m, 1H), 8.21 (t, *J* = 5.4 Hz, 2H), 7.98–7.89 (m, 3H), 7.53 (t, *J* = 5.4 Hz, 2H), 4.43 (q, *J* = 7.2 Hz, 2H), 1.44 (t, *J* = 7.2 Hz, 3H). HRMS calcd for C_22_H_16_N_2_O_4_ [M+H]^+^: 373.1183, found 373.1182.

### 4-(Propoxycarbonyl)phenyl phenazine-1-carboxylate (**5m**)

Yellow solid; yield: 98.1%; m.p. 95 °C; ^1^H-NMR (600 MHz, CDCl_3_) δ 8.61–8.40 (m, 2H), 8.41–8.30 (m, 1H), 8.31–8.27 (m, 1H), 8.27–8.15 (m, 2H), 8.02–7.82 (m, 3H), 7.64–7.46 (m, 2H), 4.33 (t, *J* = 6.6 Hz, 2H), 1.89–1.79 (m, 2H), 1.07 (t, *J* = 7.2 Hz, 3H). HRMS calcd for C_23_H_18_N_2_O_4_ [M+H]^+^: 387.1339, found 387.1340.

### 4-(Butoxycarbonyl)phenyl phenazine-1-carboxylate (**5n**)

Yellow solid; yield: 97.5%; m.p. 119–120 °C; ^1^H-NMR (600 MHz, CDCl_3_) δ 8.52–8.44 (m, 2H), 8.36–8.31 (m, 1H), 8.30–8.25 (m, 1H), 8.23–8.18 (m, 2H), 7.96–7.89 (m, 3H), 7.55–7.51 (m, 2H), 5.33–5.28 (m, 1H), 1.41 (d, *J* = 6.6 Hz, 6H). HRMS calcd for C_23_H_18_N_2_O_4_ [M+H]^+^: 387.1339, found 387.1340.

### 4-(Butoxycarbonyl)phenyl phenazine-1-carboxylate (**5o**)

Yellow solid; yield: 99.0%; m.p. 89–90 °C; ^1^H-NMR (600 MHz, CDCl_3_) δ 8.53–8.39 (m, 2H), 8.36–8.31 (m, 1H), 8.29–8.25 (m, 1H), 8.24–8.19 (m, 2H), 7.96–7.87 (m, 3H), 7.56–7.51 (m, 2H), 4.38 (t, *J* = 6.6 Hz, 2H), 1.88–1.76 (m, 2H), 1.57–1.48 (m, 2H), 1.02 (t, *J* = 7.2 Hz, 3H). HRMS calcd for C_24_H_20_N_2_O_4_ [M+H]^+^: 401.1496, found 401.1497.

### 4-(Octyloxycarbonyl)phenyl phenazine-1-carboxylate (**5p**)

Yellow solid; yield: 97.1%; m.p. 57–59 °C; ^1^H-NMR (600 MHz, CDCl_3_) δ 8.60–8.40 (m, 2H), 8.43–8.31 (m, 1H), 8.31–8.24 (m, 1H), 8.25–8.18 (m, 2H), 8.02–7.85 (m, 3H), 7.63–7.46 (m, 2H), 4.36 (t, *J* = 6.6 Hz, 2H), 1.88–1.76 (m, 2H), 1.53–1.43 (m, 2H), 1.42–1.26 (m, 8H), 0.91 (t, *J* = 6.6 Hz, 3H). HRMS calcd for C_28_H_28_N_2_O_4_ [M+H]^+^: 457.2122, found 457.2123.

### Biological assays

Compounds were screened for their in vitro fungicidal activity against *Rhizoctonia solani*, *Fusaium graminearum*, *Altemaria solani*, *Fusarium oxysporum*, *Sclerotinia sclerotiorum and Pyricularia oryzae* with the mycelium growth rate test.

The method for testing the primary biological activity was performed aseptically with pure cultures. Synthesized compounds were dissolved in 100% acetone, and the solutions were diluted with aqueous 1% Tween 80 and were then added to sterile potato dextrose agar (PDA). The target final concentration of each compound was 50 μg/mL. The control blank assay was performed with 1 mL of sterile water. Mycelial plugs 6 mm in diameter were obtained with a cork borer and placed on the amended PDA. The culture plates were incubated at 28 °C. The diameter of the mycelia was measured after 72 h. Acetone in sterile aqueous 1% Tween 80 served as the negative control, whereas phenazine-1-carboxylic acid served as positive controls. Each sample was screened with three replicates, and each colony diameter of the three replicates was measured four times. All statistical analysis was performed using EXCEL 2010 software. The log dose–response curves allowed determination of the EC_50_ for the bioassay using probit analysis. The 95% confidence limits for the range of EC_50_ values were determined by the least-square regression analysis of the relative growth rate (% control) against the logarithm of the compound concentration. The relative inhibition rate of the circle mycelium compared to blank assay was calculated via the following equation:

$${\text{Relative}}\;{\text{inhibition}}\;{\text{rate}}\;\left( \% \right) = \left[ {{{\left( {{\text{CK}} - {\text{PT}}} \right)} \mathord{\left/ {\vphantom {{\left( {{\text{CK}} - {\text{PT}}} \right)} {\left( {{\text{CK}} - 6\;{\text{mm}}} \right)}}} \right. \kern-0pt} {\left( {{\text{CK}} - 6\;{\text{mm}}} \right)}}} \right] \times 100\%$$

where CK is the extended diameter of the circle mycelium during the blank assay; and PT is the extended diameter of the circle mycelium during testing.

### Plant materials and fungal growth condition

Seeds of rice (Feng liang you xiang No. 1), with high rates of germination, were grown in plastic pots of 20 cm diameter and kept in a greenhouse under a temperature of 26–28 °C, with 10 plant per pot. After 4 weeks the four-leaf stage plants were used in the experiments. *Rhizoctonia solani* was cultured for 4 days at 28 °C on potato dextrose agar (PDA), under aseptic conditions. Spore concentration was adjusted with sterile distilled water to 10^5^ spores/mL.

### Chemical treatment of plants

Chemical treatments of plants were carried out as described by Makandar and others [34, 35]. Briefly, a stock solution of 10 mmol/L for testing conjugate **5c** (highest fungicidal activity against *Rhizoctonia solani*) was prepared in water and diluted to a final concentration of 200 μmol/L. Rice plants at the four-leaf of the similar size were sprayed with a concentration of 200 μmol/L of test conjugate **5c**, PCA and of salicylic acid (SA). A blank water control was also applied under the same conditions. There were four treatments as follows: (1) PCA, (2) SA, (3) conjugate **5c**, and water-treated control. Each treatment consisted of three pots each containing 10 rice seedlings, and were arranged in a completely randomized design and replicated four times. In all treatments, spraying was done 24 h prior to inoculation.

### Fungal inoculation and disease rating

Plants were treated with chemicals and 24 h later, point inoculations of rice leaf sheaths were done with needle injection of 10 μL of the 10^5^ spores/mL suspension at the four-leaf stage of seedlings of rice. For each replication of each treatment, 30 leaf sheaths were inoculated. The inoculated plants were covered with black plastic bags and kept in a growth room maintained at 90% relative humidity near 90% at 26–28 °C for 24 h. Plants were evaluated for rice sheath blight disease as percent leaf sheath infected with *Rhizoctonia solani* at 14 days after inoculation. All statistical analyses were performed using EXCEL 2010 software. The disease reduction was calculated as follows:

$${\text{Disease}}\;{\text{reduction}}\;\left( \% \right) = \left[ {{{\left( {{\text{CK}} - {\text{PT}}} \right)} \mathord{\left/ {\vphantom {{\left( {{\text{CK}} - {\text{PT}}} \right)} {\text{CK}}}} \right. \kern-0pt} {\text{CK}}}} \right] \times 100\%$$

where CK is the percent disease in inoculated plants treated with water while PT is the disease rating for inducer treatments.

## Conclusions

In summary, we prepared 16 novel hydroxybenzoic acid ester conjugates of phenazine-1-carboxylic acid and investigated their biological activity. Most of the synthetic conjugates displayed some level of fungicidal activity in vitro against five phytopathogenic fungi. In particular, nine conjugates **5b**, **5c**, **5d**, **5e**, **5h**, **5i**, **5m**, **5n** and **5o** (EC_50_ values were between 3.2 μg/mL and 14.1 μg/mL) were more active than PCA (EC_50_ value was 18.6 μg/mL) against *Rhizoctonia solani*, and conjugate **5c** had the highest fungicidal activity, 6.5-fold greater than PCA. The results of the bioassay indicated that the fungicidal activity of conjugates is associated with their LogP, and the optimal LogP values of the more potent fungicidal activity within these conjugates ranged from 4.42 to 5.08. The test of systemic acquired resistance against rice sheath blight disease in rice seedlings revealed that PCA–SA ester conjugate **5c** retains the resistance induction activity of SA to rice sheath blight, and has higher activity than SA. Meanwhile, the mechanism of systemic acquired resistance against rice sheath blight in rice seedlings by PCA–SA ester conjugate **5c** will be the focus of our next study.

## Additional file

**Additional file 1.** Spectrum data of PCA derivatives. Which includes the copies of 1H NMR and HRMS of selected compounds.
