# Supplementary material for: Design, synthesis and biological activity of hydroxybenzoic acid ester conjugates of phenazine-1-carboxylic acid
Source: Chem Cent J. 2018 Nov 1;12:111. doi: 10.1186/s13065-018-0478-2 (PMC6768031; doi:10.1186/s13065-018-0478-2)
Supplement: Supplementary file 1 — Additional file 1. Spectrum data of PCA derivatives. Which includes the copies of 1H NMR and HRMS of selected compounds. [file 13065_2018_478_MOESM1_ESM.doc]

Additional file 1

**Design, synthesis and biological activity of hydroxybenzoic acid ester conjugates of phenazine-1-carboxylic acid**

Xiang Zhu^1, 2^, Linhua Yu^2^, Min Zhang^2^, Zhihong Xu^2^, Zongli Yao^2^, Qinglai Wu^2^, *, Xiaoying Du^2, 3^,*, Junkai Li^1, 2^,*

*^1^ Hubei Collaborative Innovation Centre for Grain Industry, Yangtze University, Jingmi Road 88, Jingzhou 434025, China*

*^2^ School of Agriculture, Yangtze University, Jingmi Road 88, Jingzhou 434025, China*

*^3^ Engineering Research Center of Ecology and Agricultural Use of Wetland, Ministry of education, Yangtze University, Jingmi Road 88, Jingzhou 434025, China*

*E-mail address*: [cjdxnxyzx@sina.com (](mailto:cjdxnxyzx@sina.com (Qinglai)Xiang Zhu).

*E-mail address*: [linhuayu531@sina.com (](mailto:linhuayu531@sina.com ()Linhua Yu).

*E-mail address*: 249003217@qq.com [(](mailto:cjdxnxyzx@sina.com (Qinglai)Min Zhang).

*E-mail address*: [969467196@ qq.com (](mailto:969467196@sina.com ()Zhihong Xu).

*E-mail address*: [806032526@qq.com (](mailto:806032526@qq.com ()Zongli Yao).

^*^Corresponding author. Tel/Fax: +86 716-8066541.

*E-mail address*: [wql106@163.com (Qinglai](mailto:wql106@163.com  (Qinglai) Wu).

^*^Corresponding author. Tel/Fax: +86 716-8066314.

*E-mail address*: Qinger539@163.com (Xiaoying Du).

^*^Corresponding author. Tel/Fax: +86 716-8066767.

*E-mail address:* junkaili@sina.com (Junkai Li).

**^1^H NMR and HRMS spectra**

**1. Spectra of compound 5a**

**^1^H-NMR Spectrum**

**HRMS Spectrum**

**2. Spectra of compound 5b**

**^1^H-NMR Spectrum**

**HRMS Spectrum**

**3. Spectra of compound 5c**

**^1^H-NMR Spectrum**

**HRMS Spectrum**

**4. Spectra of compound 5d**

**^1^H-NMR Spectrum**

**HRMS Spectrum**

**5. Spectra of compound 5e**

**^1^H-NMR Spectrum**

**HRMS Spectrum**

**6. Spectra of compound 5f**

**^1^H-NMR Spectrum**

**HRMS Spectrum**

**7. Spectra of compound 5g**

**^1^H-NMR Spectrum**

**HRMS Spectrum**

**8. Spectra of compound 5h**

**^1^H-NMR Spectrum**

**HRMS Spectrum**

**9. Spectra of compound 5i**

**^1^H-NMR Spectrum**

**HRMS Spectrum**

**10. Spectra of compound 5j**

**^1^H-NMR Spectrum**

**HRMS Spectrum**

**11. Spectra of compound 5k**

**^1^H-NMR Spectrum**

**HRMS Spectrum**

**12. Spectra of compound 5l**

**^1^H-NMR Spectrum**

**HRMS Spectrum**

**13. Spectra of compound 5m**

**^1^H-NMR Spectrum**

**HRMS Spectrum**

**14. Spectra of compound 5n**

**^1^H-NMR Spectrum**

**HRMS Spectrum**

**15. Spectra of compound 5o**

**^1^H-NMR Spectrum**

**HRMS Spectrum**

**16. Spectra of compound 5p**

**^1^H-NMR Spectrum**

**HRMS Spectrum**
